# Responses of soil respiration to nitrogen addition in the Sanjiang Plain wetland, northeastern China

**DOI:** 10.1371/journal.pone.0211456

**Published:** 2019-01-31

**Authors:** Jianbo Wang, Xiaoling Fu, Zhen Zhang, Maihe Li, Hongjie Cao, Xiaoliang Zhou, Hongwei Ni

**Affiliations:** 1 Institute of Natural resources and Ecology, Heilongjiang Academy of Sciences, Harbin, Heilongjiang, China; 2 Northeast Forestry University, Harbin, Heilongjiang, China; 3 Swiss Federal Research Institute WSL, Birmensdorf, Switzerland; 4 Institute of Applied Ecology, Chinese Academy of Sciences, Shenyang, Jilin, China; 5 The Honghe National Nature Reserve, Jiansanjiang, Heilongjiang, China; Beijing Normal University, CHINA

## Abstract

This study was designed to test the hypothesis that nitrogen (N) addition leads to enhanced soil respiration (SR) in nitrogen deficient marsh. Here, we report the response of SR to simulated N deposition in a temperate marsh of northeastern China from June 2009 to September 2011. The experiment included three-levels of N treatment (control: no N addition, Low-N: 4g N m^-2^ y^-1^, and High-N: 8 g N m^-2^ y^-1^). Our study showed various responses of SR to level and duration of N addition. Yearly SR was increased by 11.8%-15.2% (P<0.05) under Low-N addition during the three years, while SR showed a strong increase by 27.5% (P<0.05) in the first year and then decreased by 4.4% (P>0.05) and 15.4% (P<0.05) in the next two years under High-N addition. Soil respiration was positively correlated with soil temperature and negatively correlated with soil water content. High-N treatment reduced soil pH value (P<0.05). The negative response of SR to High-N addition in the following two years may attribute to lower microbial activity, microbial biomass and alteration in the microbial community due to lower soil pH, which consequently leads to decreased SR. Meanwhile, we found root biomass were increased under High-N addition. This implies that the increase of autotrophic respiration was lower than the decline of heterotrophic respiration in the following two years. Our findings suggest complex interactions between N deposition and SR, which is needed to be further investigated in the future studies.

## Introduction

Human activities such as fossil fuel combustion and fertilizer production have been enhancing the N deposition [1,2]. It is estimated that 200 Tg N yr^-1^ will be emitted and then deposited to the Earth’s surface by 2050 [3]. In Asia, reactive N deposition increased from 14 Tg N yr^-1^ in 1961 to 68 Tg N yr ^-1^ in 2000 and is expected to reach 105 Tg N yr^-1^ in 2030 [4]. Currently this leads to high atmospheric N deposition (NH_4_^+^ -N, NO_3_^-^ -N), thus causing N saturation to an extent and influencing terrestrial ecosystems by altering the soil N availability. In general, soil N availability affects plant growth, net primary productivity in N-deficient terrestrial ecosystems. As a result, N deposition can influence root respirations [5,6], microbial composition and activities [7–9], the decomposition rate of soil organic matter (SOM) [10]. It has been reported that N deposition can affect the CO_2_ exchange between the biosphere and atmosphere, thus potentially causing positive or negative feedbacks to future climate [11,12]. It is, therefore, significant to address the effects of N deposition on the fluxes of CO_2_.

Soil respiration (SR) is one of the largest C flux between terrestrial ecosystems and atmosphere, consists of root respiration and microbial respiration. Numerous modeling showed that a small amount of change in SR has significant influences on the global CO_2_ budget [13].

Experimental studies indicated that the responses of SR to N addition are variable and complex, because N addition could alter plant above- and belowground biomass and their ratios, litter quantity and quality, soil microorganisms [14–17]. Moreover, changes in the relative contribution of root respiration to SR could also be an important factor in affecting SR to different levels of N addition [18]. In general, most studies have showed that SR was stimulated by relatively Low-N additions, particularly in N-limited ecosystems [19–21]. In contrast, high-N addition have less consistent effects on SR, [22–24]. Also, Ren et al. [25] reported that SR increased in the first year and then decreased in an alpine grassland, because N additions might initially stimulate soil microbial activity thus increase SR, but over time would result in a carbon-limited state after combined plant and microbial demand for N was satisfied [26]. However, the induced C limitation to soil microorganisms would lead to the inhibition of microbial activity and thus decrease soil heterotrophic respiration and SR when N addition over a long period [27,28]. Overall, the levels and duration N addition maybe a dominant driver of different responses of SR to N addition.

The effect of N addition on SR has been widely studied in forest ecosystems [12,14,17,23,24], agricultural ecosystems [29–31] and grassland ecosystem [13,18,19,21,22,25]. However, there is a lack of report for wetland ecosystems [32,33]. Wetlands cover 6% of the world’s land surface, but hold about 12% of the global carbon pool, and play an important role in global carbon cycle [34, 35]. The Sanjiang Plain wetland is 1.04×10^4^ km^2^ in areas in northeast China, a low floodplain was formed by the Heilongjiang River, the Songhuajiang River and the Wusulijiang River. The Sanjiang Plain is N deficient, as indicated by the strong stimulation of gross primary productivity after N addition [36]. As the Sanjiang Plain wetlands impacted by intensive cultivations over past 50 years [37], the Sanjiang Plain wetlands receive more N due to inputs from the agricultural activities. Thus, investigating the changes in the net C balance of the wetland ecosystem therefore has important implications for the regional C balance.

Here, we conducted a manipulative experiment *in situ* with low and high level of N addition in a wetland in China from 2009 to 2011. We hypothesize that (1) low-N addition should increase SR, while high-N addition should decrease SR and (2) The response of root biomass and soil abiotic factors to N addition should highly related with SR.

## Materials and methods

### Study site

This study was conducted in the Honghe National Nature Reserve (47°49′N, 133°40′E), a temperate wetland in Heilongjiang Province, northeastern China. The Honghe National Nature Reserve is a N deficient marsh dominated by perennial grass species *Calamagrostis angustifolia* Kom. with plant coverage of 90%. The study site is temperate continental monsoon climate with the annual temperature of 2.5°C and the mean annual precipitation of 500–650 mm (80% of which fall in summer), and frost-free period of 120–140 days. Meadow soil is the main soil type with 20% of soil organic matter content and 0.5% of total N at a 0–10 cm soil depth [38].

### Experimental design and ethics statement

In May 2009, nine rarely-flooded plots (20 m×20 m each) were established along the wetland edge. We randomly selected three plots for ambient nitrogen (control, i.e. no nitrogen is added), three for low nitrogen addition (Low-N, 4g N m^-2^ y r^-1^), and three for high nitrogen addition (High-N, 8g N m^-2^ y r^-1^). Over 10 m buffer stripes were set up between any two adjacent plots. The plots were fertilized on 20 May 2009, 22 May 2010, and 18 May 2011, respectively, in the form of NH_4_NO_3_ solution using backpack sprayers. The same amount of water was applied to the control plots.

All work was undertaken with relevant permissions from the Honghe National Nature Reserve, China for our observational and field studies. The field studies did not involve endangered or protected species.

### Measurements of soil respiration, root biomass, soil dehydrogenase activity (*DHA*) and soil pH

From June 2009 to September 2011, SR were measured using a Li-COR 6400 infrared gas analyzer connected with a 6400–09 soil respiration chamber (Li-COR, Inc., Lincoln, NE, USA) during the growing seasons (May-September). Five soil collars (8 cm in diameter and 5 cm in height) were randomly distributed and installed into the soil (3 cm depth) in each plot in May 2009. The soil collar matched well with SR chamber to avoid possible leakage. Collars remained in the same measurement locations throughout the experiment period. SR was measured between 9:00 a.m. and 11:00 a.m. (local time). Soil temperatures (T_soil_) at 5 cm depth (Soil Temperature Probe 6400–09 TC, Li-Cor Inc., Lincoln, NE, USA) were recorded near each collar while SR was being measured. Soil water content (SWC) at 5 cm depth was continuously measured with an ECH_2_O dielectric aquameter (EM50/R Decagon Ltd, Pullman, WA, USA) during the experiment period.

Root biomass was estimated for each plot by using the root in-growth method [22]. We excavated three 30-cm-deep cylindrical holes using a 10-cm diameter soil auger in each plot in early May of each year. The soils were refilled to the same hole after removing root via 2-mm sieves. In the late September of each year, we collected root in-growth samples at the center of the original root-in-growth holes. Root samples were weighted after washing and oven-drying at 70°C at least for 24 h.

Soil cores were taken randomly from 3 points at a depth of 0–10 cm in the plots in September 2011. After removal of debris from plants and animals, soil samples were divided into two parts, sealed in plastic bags in a cooler and transported to the laboratory for further treatment. One aliquot of each sample (the field-moist samples) were sieved (2 mm mesh) and stored in sealed plastic bags at 4°C for *DHA* analysis. *DHA* was determined by the reduction of triphenyltetrazolium chloride (TTC) to triphenylformazan (TPF). Specifically, moist soil was treated with 2.5 ml of 1% TTC-Tris buffer and incubated at 37°C in darkness for 24 h [39]. Data are expressed as μgTPFg^-1^ dry soil 24h^-1^. *DHA* activities were analyzed as indicative measures of soil microbial activity. One aliquot was air dried, thoroughly grinded and passed through sieves (1.0 mm). The sieved samples were used to determine the soil pH. Soil pH was measured in suspension with a water-to-soil ratio of 2.5:1.

### Statistical analysis

Repeated measures ANOVAs were performed to examine the effects of N addition on SR, T_soil_, and SWC for the period between June 2009 and September 2011. One-way ANOVA with Tukey’s HSD test was used to test the difference among treatments on root biomass, soil pH, and soil *DHA*. Correlation and nonlinear regression analyses were used to explore the relationships between soil temperature or soil water content and SR. We fitted measured soil temperature and SR to the exponential equation (*R* = α*e*^β*T*^) and obtained the *Q*_10_ value from the β coefficient (*Q*_10_ = *e*^10β^). One-way ANCOVA test was used to compare the regression slopes among treatments. All analyses were performed with SPSS 13.0 software package (SPSS Inc., Chicago, IL, USA), and graphs were prepared with SigmaPlot 11.0 software (Systat Software Inc., Chicago, IL, USA).

## Results

### Soil microclimate changes induced by N addition treatments

The average T_soil_ of 2009 was significantly higher than that of 2010 and 2011 (P<0.001, Tables 1 and 2). N fertilization showed notable effects on the T_soil_ and SWC. Low-N and High-N addition significantly decreased T_soil_ by 3.4% and 3.9%, respectively (P<0.05). Similarly, SWC was higher in Low-N and High-N addition than in the control plots (P<0.05, Table 2).

**Table 1:** Growing season means of soil temperature (T_soil_,°C), soil water content (SWC) and soil respiration (SR, μmolm^−2^s^−1^) under different treatments in 2009, 2010 and 2011.

Year	Treatment	T_soil_	SWC	SR
	**CK**	16.45±0.2	44.81±2.3	6.58±0.16
**2009**	**Low-N**	16.04±0.3	44.00±1.8	7.36±0.23
	**High-N**	16.00±0.2	42.80±2.1	8.39±0.14
**2010**	**CK**	16.08±0.3	44.27±2.2	5.18±0.13
	**Low-N**	15.24±0.4	42.24±2.1	5.84±0.14
	**High-N**	15.13±0.3	42.04±1.6	4.96±0.21
**2011**	**CK**	15.52±0.3	51.98±1.8	4.74±0.13
	**Low-N**	15.09±0.2	50.23±1.7	5.46±0.16
	**High-N**	15.05±0.4	50.86±1.3	4.01±0.11

Values represent the Means± SE.

**Table 2:** Results (*P* value) of repeat-measurement ANOVA on the effects of year, N addition (N) and their interactions on soil temperature (T_soil_), soil water content (SWC), and soil respiration (SR).

Source of variation	df	*P* values		
		T_soil_	SWC	SR
**Year**	2	<0.001	0.001	<0.001
**N**	2	<0.01	<0.05	<0.01
**Year×N**	4	<0.01	<0.01	<0.01

### Seasonal and inter-annual variations of soil respiration

**Fig 1:**
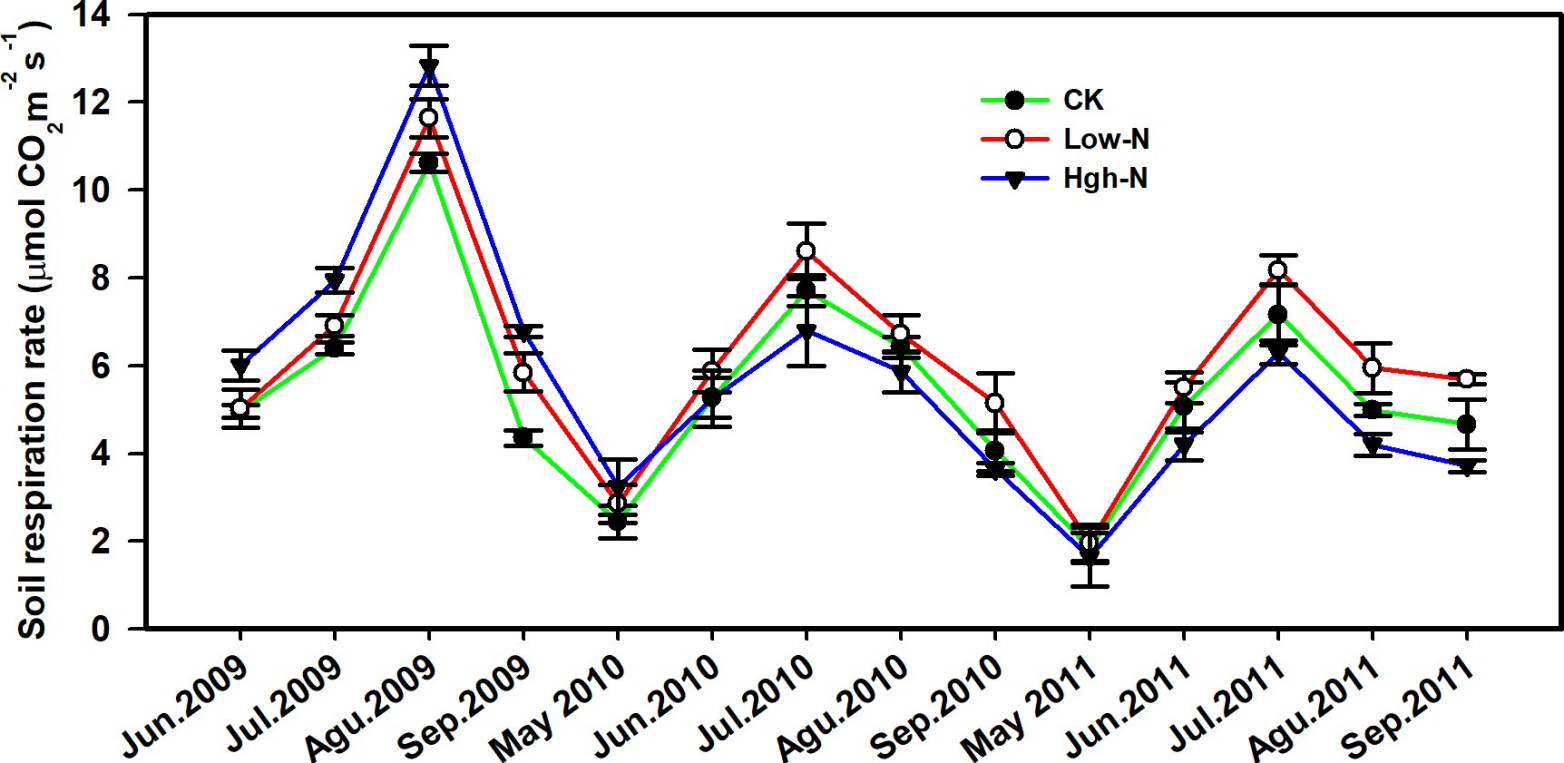
Seasonal dynamics of soil respiration rate in relation to N treatment during 2009–2011.

SR showed a parabolic-like pattern with the maximum values in August or July (Fig 1). SR was significantly higher in 2009 than in 2010 and 2011 (Tables 1 and 2; P <0.001). In the control plots, seasonal means for SR in 2009 was 21% and 30% greater than in 2010 and 2011, respectively. The remarkable inter-annual variability in the SR might be partly attributed to the difference in soil temperature.

### Effects of N addition on soil respiration

**Fig 2:**
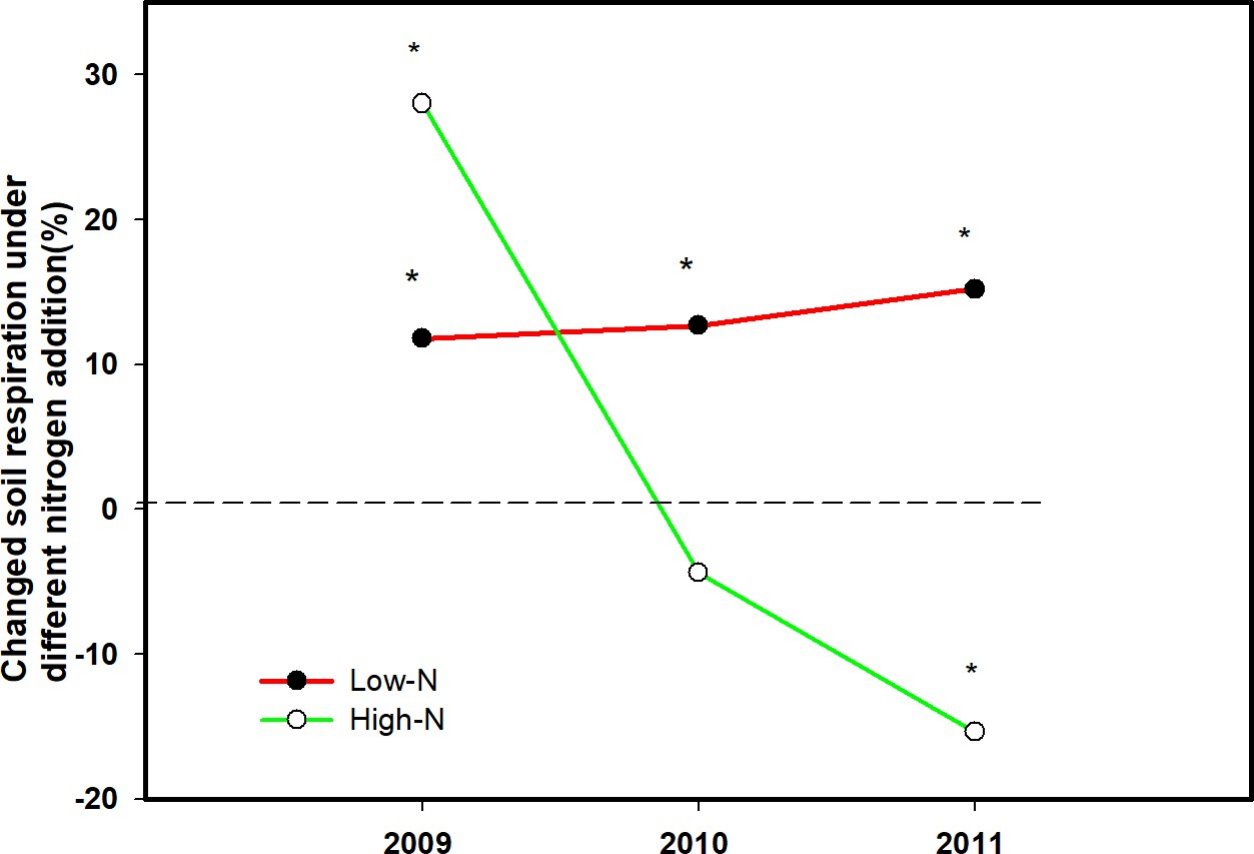
Effects of different nitrogen treatments on changes (%) of soil respiration during the growing seasons from 2009 to 2011.

N addition had a significant effect on SR, where the significant correlation between N addition and the year were observed (P<0.01 for the N, N×year, Table 2). Specifically, Low-N treatment enhanced soil respiration by 11.8%, 12.7% and 15.2% in 2009, 2010 and 2011, respectively (P<0.05, Figs 1 and 2). However, High-N treatment was found to increase SR by 27.5% in 2009 (P<0.05), and reduced SR by 4.4% (P>0.05) and 15.4% (P<0.01) in 2010 and 2011, respectively (Figs 1 and 2). Among the three growing seasons, CK plots had the lowest SR (5.50 ± 0.55 μmol CO_2_ m^-2^ s^-1^), followed by the High-N plots (5.78± 1.33 μmol CO_2_ m^-2^ s^-1^), and Low-N plots (6.22 ± 0.58 μmol CO_2_ m^-2^ s^-1^).

### Effects of N addition on root biomass, soil pH and *DHA*

**Fig 3:**
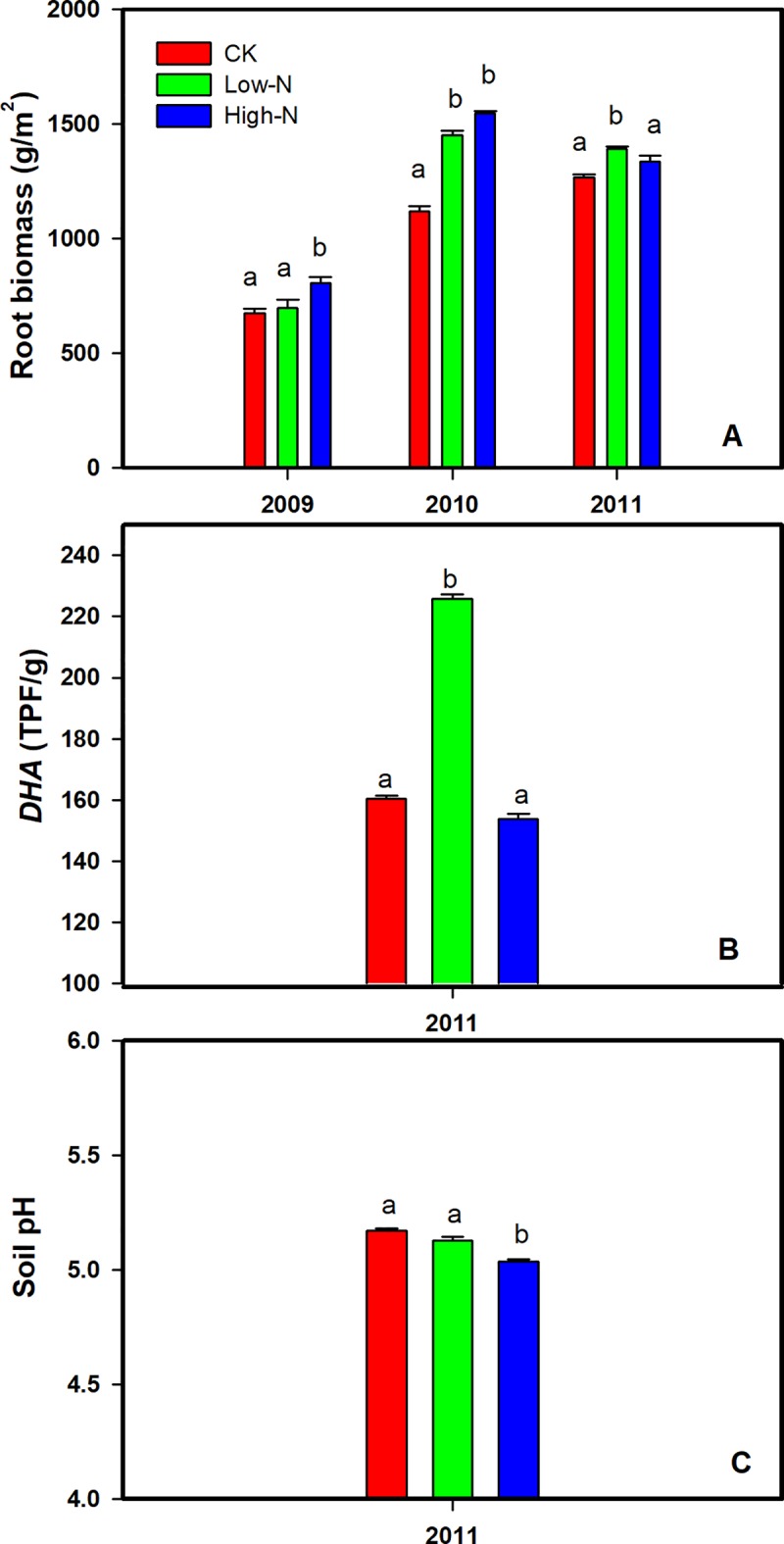
Effects of N addition on root biomass (A), soil dehydrogenase activity (*DHA*) (B), and soil pH (C).

N addition showed significant effects on the root biomass with varied correlation across the 3 yr. High-N treatment significantly increased root biomass by 19.1% (P<0.05) in 2009 compared to CK. Low-N and High-N treatment enhanced root biomass by 29.7% and 38.5% (P<0.05), respectively in 2010 (Fig 3A). However, root biomass was only higher in Low-N treatment compared with that in the control plots in 2011.

After 3 years of N treatment, Low-N treatment significantly increased soil *DHA* by 40% (P < 0.01), however, High-N treatment slightly reduced soil *DHA* by 4.1% (P = 0.06, Fig 3B). High-N treatment significantly reduced soil pH value by 2.7% (P<0.05, Fig 3C).

### Dependence of soil respiration on biotic and abiotic factors

**Fig 4:**
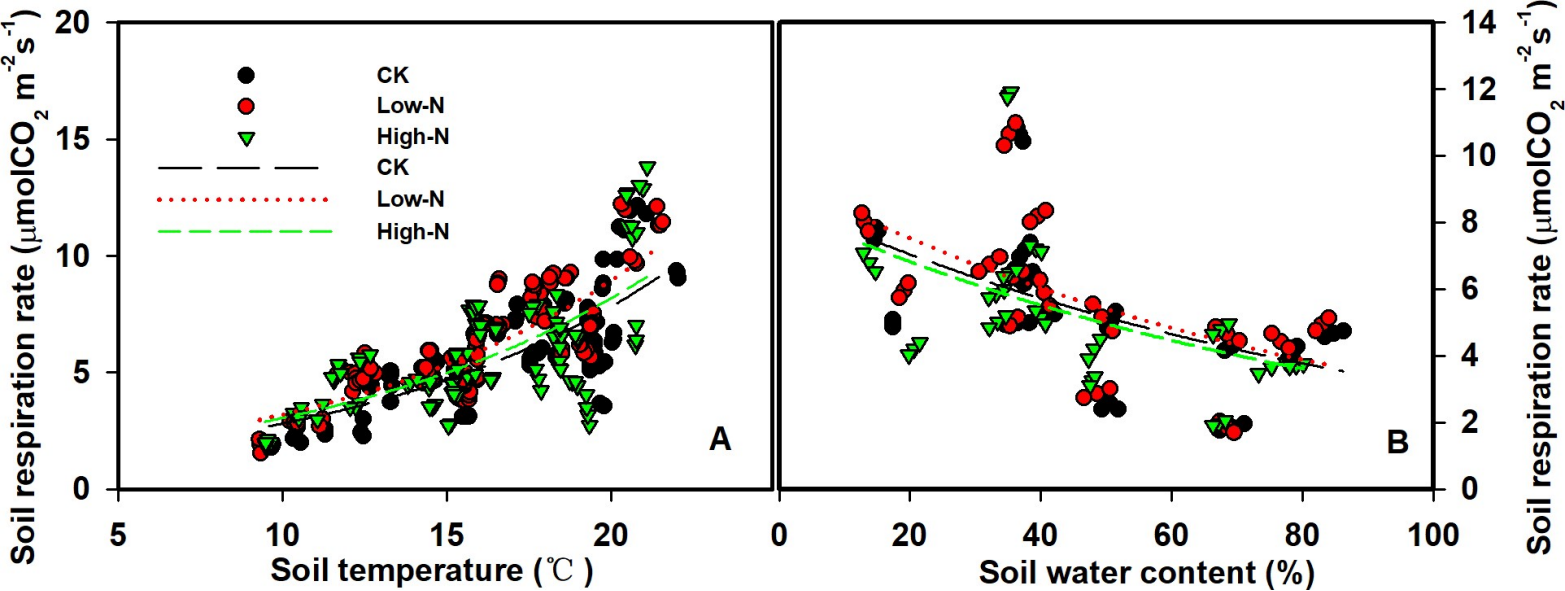
Relationships between soil temperature (°C,A), soil water content (%, B) at 5 cm depth and soil respiration rate.

**Fig 5:**
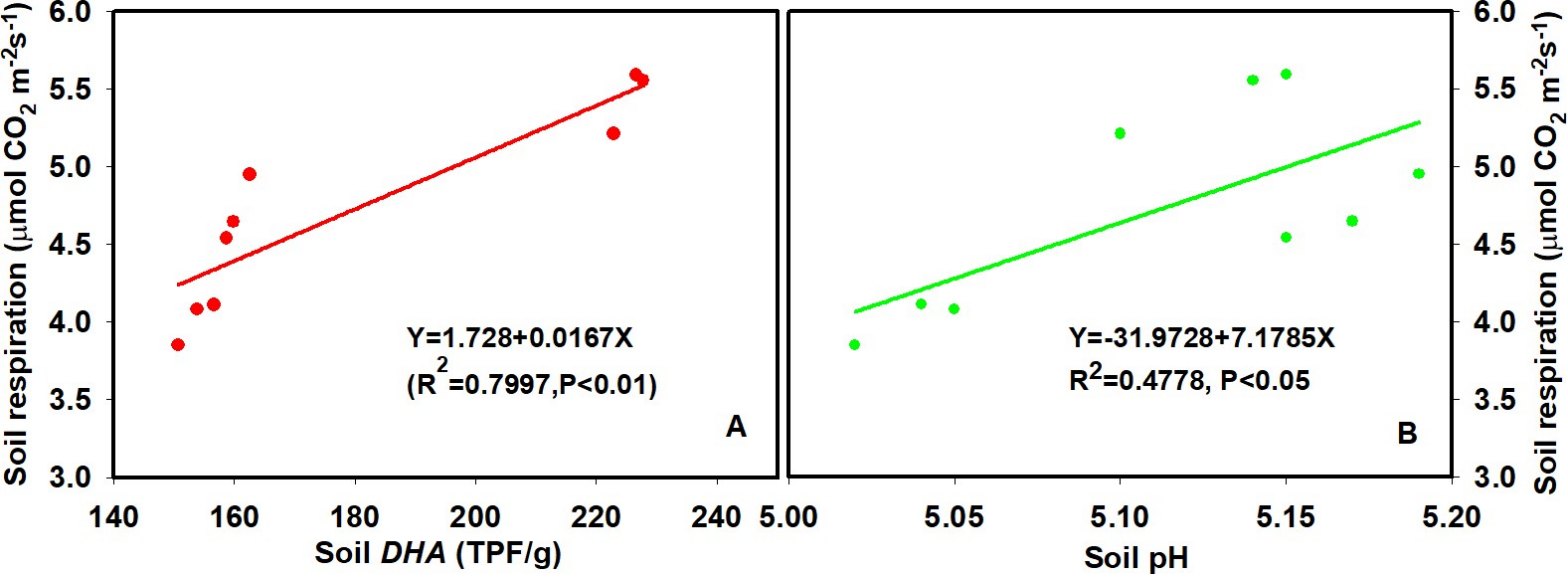
Relationships between soil *DHA* (A), soil pH (B) and soil respiration rate.

SR exhibited significant positive exponential relationships with T_soil_ in all treatments (R^2^ = 0.45–0.68, all p <0.0001, Fig 4A, Table 3). N addition had no significant effect on the temperature sensitivity of SR (*Q*_10_ value, Table 3). The highest value of *Q*_10_ occurred in the Low-N treatment at 2.81, and the lowest value occurred in the High-N treatment at 2.69. Negative exponential relationships were found between SR and SWC over the three years period in all treatments (R^2^ = 0.23–0.34, all p<0.01; Fig 4B, Table 4). Moreover, the relationships between soil respiration rate and soil *DHA* and soil pH were found were significant and linear (p<0.05, Fig 5).

**Table 3:** Models for relationships between soil temperature and soil respiration under different N treatments.

Treatment	α	β	*Q*_10_	R^2^	*P*
**CK**	1.0042	0.1026	2.79 a	0.61	<0.0001
**Low-N**	1.1282	0.1035	2.81 a	0.68	<0.0001
**High-N**	1.1325	0.0988	2.69 a	0.45	<0.0001

**Table 4:** Models for relationships between soil water content and soil respiration under different N treatments.

Treatment	a	b	R^2^	*P*
**CK**	8.6698	0.0104	0.29	<0.01
**Low-N**	9.3743	0.0110	0.34	<0.001
**High-N**	8.4341	0.0107	0.23	<0.01

## Discussion

The large seasonal variations in SR were similar in the control and N addition plots during the experiment. The strong seasonal pattern of SR has also been reported in many other studies, such as a temperate steppe, grassland [40–42] and forest [23,24,43]. We found positive exponential relationships with T_soil_ in all treatment, as well as negative exponential relationships between SR and SWC, which was consistent with one previous study [23]. In our study, T_soil_ and SWC explained 50%-69% and 23%-34% variations of soil CO_2_ flux, respectively.

Consistent with our first hypothesis, Low-N addition has a constant positive effect on SR during the three years, while High-N addition significantly stimulated SR during the growing season in the first year of the experiment, and then decreased SR in the following two years. The different responses of SR to different N level over time highlight the importance of conducting the long-term field experiment.

The results in the first year were in line with a wide range of results reported in previous studies for SR responses to N additions in other ecosystems. For example, increased SR agrees well with results for temperate grasslands [42] and coastal marshes in New England [33]. The gross primary productivity in the Sanjiang Plain marsh has high sensitivity to N addition because of its N-deficient environment [36].Therefore, when N was initially added in the first year, increased N availability stimulated plant growth and plant biomass, which could potentially lead to higher autotrophic respiration [44,45]. On the other hand, carbon input from additional aboveground litter and decomposed roots finally entered into the soil organic matter and would stimulate heterotrophic respiration [23, 46].

In the following two years, Low-N addition continued to increase SR, however, High-N levels decreased soil respiration. Similarly, He et al. [47] and Cao et al. [48] found that a low level of N addition enhanced SR and high-level N addition inhibited SR in the *Populus euphratica* and *Acacia mangium* forest, respectively. They concluded that the divergent responses of SR to the different N addition may be related to the N status of soil [49]. The exact reason for the negative effect in the High-N treatment in our results has not yet been identified, but it can be explained by three possible mechanisms: (1) High-N additions initially significantly stimulated SR in the first year and consumed greater C substrates. Consequently, the study site would be exposed to a carbon-limited state. Micks et al. [50] found that N addition seldom influenced microbial respiration in a C-limited condition. (2) External N addition could inhibit or interfere with enzymes involved in decomposition [51]. Moreover, This inhibitory effect was strongest where added N greatly exceeds ambient deposition [52], which might explain why SR decreased in the high N treatment. (3) The variation of soil pH plays an important role in the decomposition of soil organic matter [53]. Reduced soil pH value under High-N treatment (Fig 3C) may inhibit microbial activities [23,54]. Additionally, declines in soil *DHA* activity (P = 0.06) in our study may also dampen soil microbial activities in the High-N treatment. Although we have no direct experimental measurements for the soil microbial activities, Garcia et al. [55] have found that *DHA* is a good indicator of the status of soil microbial activity. Moreover, Sui et al. found that High-N addition decreased soil fungal diversity and affected the fungal community composition and the relative abundance of species in the same experiment site and treatment [56]. Therefore, the alterations in the microbial community may also affect enzyme production and decomposer efficiency, and thus decrease respiration rate [23]. In addition, Compton et al. [57] and Frey et al. [7] observed that N addition reduced microbial biomass and the active fungal biomass. Consequently, soil heterotrophic respiration may be suppressed by declined microbial activities, microbial biomass, and the alteration in the microbial community in the High-N plots.

Importantly, given that High-N treatment increased root biomass (Fig 3A) and thus increased autotrophic respiration to some extent, our results suggest that the increased autotrophic respiration contributed to the total SR was lower than the heterotrophic respiration. Thus the increase of autotrophic respiration was insufficient to balance the decline of heterotrophic respiration, which consequently decreased the total soil CO_2_ flux in the High-N plots.

The temperature sensitivity of SR (*Q*_10_) is considered as an important indicator for assessing thermal acclimation of SR [58,59]. The mean *Q*_10_ in our study was 2.69–2.81 (Table 3), which is within the range of global average value (1.3–3.3) [60]. In our study, there was no obvious acclimation of SR under Low-N and High-N treatment, which is consistent with a study in the grassland where low-N and high-N treatments have no effect on *Q*_10_ [42]. Our results revealed that nitrogen addition changed SR but has little effect on the temperature sensitivity of SR.

## Conclusions

We find that soil respiration exhibited a strong seasonal pattern, with the highest rates observed during the summer (June-August) and the lowest rates during in the spring and autumn. Both soil temperature and soil water content were dominant factors on soil respiration in our study. Our results found that different responses of SR to level and duration of N addition: High-N addition significantly stimulated SR during the growing season in the first year of the experiment, and decreased SR in the following two years; however, Low-N addition continued enhancing SR rate during the three years. The negative effect in High-N plots on SR in the following two years may be attributed to lower microbial activity, lower microbial biomass and the alteration in the microbial community, which consequently inhibited heterotrophic respiration. This implies that the contribution of the autotrophic respiration was lower than the heterotrophic respiration in High-N addition treatment. Our results suggest that response of soil respiration to N deposition could be significantly influenced by the magnitude of N deposition in the temperate wetland. This requires further long-term experiments to fully quantify the consequences of nitrogen deposition on soil respiration, especially with a focus on the response to high nitrogen deposition.

## Supporting information

S1 File Minimal data for this study. (XLSX)
